# Ethological, Clinical, and Neurobiological Studies on Cannibalism in Black-Necked Pheasants (*Phasianus colchicus*) and Correction of Behavioral Disorders by Applying Nutritional Supplements

**DOI:** 10.3390/ani15243561

**Published:** 2025-12-11

**Authors:** Slavko Nikolov, Rositsa Mileva, Antoaneta Yordanova, Nadya Bozakova, Aneliya Milanova, Dian Kanakov

**Affiliations:** 1 Faculty of Veterinary Medicine, University of Forestry, 1756 Sofia, Bulgaria; 2 Faculty of Veterinary Medicine, Trakia University, 6000 Stara Zagora, Bulgaria; rositsa.mileva@trakia-uni.bg (R.M.); nadiab@abv.bg (N.B.); dian.kanakov@trakia-uni.bg (D.K.); 3 Faculty of Medicine, Trakia University, 6000 Stara Zagora, Bulgaria; antoaneta.yordanova@trakia-uni.bg

**Keywords:** damaging behavior, injurious pecking, game birds, tryptophan, silymarin, neurotransmitters, serotonin, dopamine

## Abstract

Cannibalism in Black-necked pheasants is a problem in aviary rearing, and an effective solution is still lacking. We performed the present study to investigate the potential of using tryptophan or silymarin to suppress cannibalism. The following types of investigations were performed: evaluation of the pheasants’ harmful behavior manifestations, and evaluation of the clinical forms, severity and localization of injuries of feathers and tissues, and neurohormonal manifestations. Additionally, the potential of cannibalism control in pheasants by supplementing tryptophan and silymarin to the birds’ diet was investigated. Ethological studies have shown a low intensity of feather pecking in pheasants without cannibalism manifestations. In pheasants with pronounced cannibalism, severe forms of feather and tissue pecking were observed in the head, back, wings, and rump areas, as well as specific forms affecting the tail feathers and the cloaca. Tryptophan and silymarin significantly reduced the levels of injurious pecking in the studied game birds and improved the healing of the lesions. The blood serotonin and dopamine levels in pheasants manifesting cannibalism were significantly lower than those in birds which did not show such behavior. The addition of tryptophan or silymarin to the diet of birds exhibiting cannibalism resulted in significantly increased plasma concentrations of serotonin or dopamine.

## Simple Summary

Game bird breeding is of utmost importance in hunting farms, with common pheasant, gray, and chukar partridge subspecies being the most popular European species. There are a few reasons why the industry is growing; one of them is that sport hunting is becoming increasingly popular, which necessitates a larger number of game birds to be produced for shooting. Other factors include biodiversity improvement and ecology protection in EU countries through the resettlement of these species in the wild, and the increased production of game meat and eggs for the food industry. The gathering and rearing of large numbers of pheasants in captivity in an unnatural habitat are associated with a number of restrictions. These conditions impede physiological behavior, meaning that pheasants become frustrated, and thus serious problems develop. One example of such problems is the behavioral disorders developed when birds focus their attention on the feathers or skin of other individuals in the group. This offensive behavior significantly reduces production in hunting farms and leads to the death of a great number of birds. The aim of the present study was to reduce or eliminate the cannibalism-induced damage in pheasants through the application of dietary supplements as alternative and non-invasive methods to address this problem.

## 1. Introduction

The proportion of hunting farms in Europe is increasing, for the purpose of sport hunting and shooting of game birds [1,2]. The production of meat and eggs from feathered game for human consumption is also high [3,4]. In these cases, the birds are removed from their normal habitat and placed in aviary conditions. When raising a large number of wild birds in captivity, the vital parameters [5,6] and needs of the birds are often disrupted, leading to the development of various behavioral problems [7,8,9].

The most common problem in individual wild bird species such as pheasants, partridges, grouse, wild turkeys, and guinea fowl in game farms is injurious pecking (IP), which includes feather (FP) and tissue pecking, known as cannibalism [10,11,12]. These behavioral disorders appear as a result of a combination of stimuli such as nutritional deficiencies, stress, natural foraging instincts, and environmental conditions related to high temperatures and long periods of light. The impact from an economic point of view is substantial, causing significant mortality among game birds, with pheasants being the most widespread in Europe, including Bulgaria [5,13]. Reduced production efficiency decreases the number of pheasants available for hunting, which significantly affects the income received from this expensive and high-cost sport [10,14]. Despite this problem being widespread, there are no clear criteria for characterizing the severity of cannibalism in aviary-raised birds, as well as means to fully prevent or inhibit the undesirable behavior solely by proper feeding or the addition of supplements to the diet.

The use of old and inhumane methods to combat behavioral disorders on hunting farms, such as the use of beak bits (rings and spectacles) [13], beak trimming, and blunting [14,15] or the use of reduced light intensity [16], does not meet animal welfare requirements and the EU’s recommendations to reduce the use of invasive methods. Therefore, new and alternative approaches are required to correct the damage caused by cannibalism.

A new and modern method used to help stop cannibalism is applying high levels of one of the following aromatic amino acids (AAAs) to birds’ diets: tryptophan (TRP), phenylalanine (PHE), or tyrosine (TYR) [5,9]. High doses of TRP are often added to the diet of chickens to reduce their levels of harmful behavior [10,17]. The relationship between TRP metabolism in chickens and the manifestation of severe feather pecking (SPF) has been proven [18,19]. Our previous studies revealed that administering high doses of TRP via animal feed has a positive effect on game birds exhibiting cannibalism [20,21].

The administration of milk thistle extract can be used as an alternative method to control cannibalism in pheasants. Several authors [22,23,24,25,26] have confirmed the biological activities of silymarin (SIL), such as its antioxidant, anticarcinogenic, hepatoprotective, anti-inflammatory, immunoregulatory, and anti-apoptotic effects. Our previous investigations demonstrated the positive effect of SIL on Black-necked pheasants exhibiting cannibalism by influencing oxidative parameters [20] and improving parameters related to fat metabolism in game birds [21]. SIL has a beneficial effect on quails subjected to chronic temperature stress [27], exhibits significant antioxidant activity, and improves the growth of quails [28]. Furthermore, the application of SIL in metabolic disorders related to the liver in parrots significantly improves their condition [29,30].

Therefore, the aim of the present study was to conduct ethological and clinical investigations to determine the exact type and localization of injurious pecking in Black-necked pheasants, as well as to determine the levels of serotonin (5-hydroxytryptamine, 5-HT) and dopamine (DA) in order to establish the presence of their changes in relation to behavioral disorders and the possible correction of cannibalism in pheasants by applying additives (TRP and SIL) to the feed.

## 2. Materials and Methods

Chemicals and reagents: Serotonin and DA, used for LC-MS/MS analysis of their concentrations in plasma, were obtained from Sigma-Aldrich (Sigma-Aldrich, St. Louis, MO, USA). The following reagents, of LC-MS/MS purity, were used: formic acid (LC/MS purity ~98%, Honeywell Fluka™, Seelze, Germany), methanol (CHROMASOLV^®^, LC-MS purity ≥ 99.9%, Honeywell Fluka™, Seelze, Germany), acetonitrile (CHROMASOLV^®^, LC-MS purity ≥ 99.9%, Honeywell Fluka™, Seelze, Germany), trifluoroacetic acid, and water for LC-MS chromatography (LiChrosolv^®^, Merck KGaA, Darmstadt, Germany). Feed additives amino acid L–tryptophan (TRP) was obtained from ST Island 0767-012A/PO No 20200219—VIAND/Ever Lunar 1316-022W/ BBD (PT. Cheil Jedang Indonesia, Jakarta, Indonesia). Silymarin (SIL) supplement (78% Extr. *Silybum marianum*), a light yellow powder, was obtained from Wuxi Gorunjie Technology Co., Ltd. (Xian, Jiangsu, China); it was designated as INM-7035.

Experimental design: The experiments were performed after we obtained a permit for the use of animals in experiments No. 280/24.09.2020 for wild birds kept in an aviary, issued by the Bulgarian Food Safety Agency. The main experimental work was conducted at the Southeastern State Division of the State Forestry in Stara Zagora, at the pheasant farm in the village of Trunkovo (livestock facility registration No 6266A-0207, Trunkovo, Bulgaria). The study was carried out in close collaboration with the veterinarians at the farm. The study included 80 Black-necked pheasants (*Phasianus colchicus* ssp. *colchicus*) aged 52–54 weeks. Through performing an ethological examination, the pheasants were classified as clinically healthy, denoted as the negative control group (without manifestation of IP and no plumage damage), or birds exhibiting injurious pecking, which were randomized into the positive control group and the treatment groups (all showing feather and skin damage). Thus, the birds were grouped into four groups, and each group consisted of 10 female and 10 male pheasants. The studies were conducted for a test period of 30 days (March 2021), and they were part of a large-scale study for a Ph.D. thesis. The birds were kept in an aviary with the aim of providing conditions as close as possible to their natural environment. They had access to the specified type of feed (Table S1) and fresh water ad libitum. Each bird in the study was provided with 2 m^2^ of space. The aviaries had a row of perches, a sheltered area for protection during bad weather, and a sand-filled area for dust bathing. During the study period, the average regional temperatures ranged from a maximum of 14.3 °C to a minimum of −0.3 °C. The body weight of the pheasants from the negative control group ranged from 1.148 ± 0.24 kg before the start of the experiment to 1.180 ± 0.22 kg at its end. For pheasants which were administered tryptophan, these values ranged from 0.891 ± 0.27 kg to 1.032 ± 0.18 kg, and for the birds treated with silymarin they were 0.916 ± 0.26 kg to 1.052 ± 0.23 kg. The mean body weight of the pheasants from the positive control group was 0.943 ± 0.27 kg at the beginning of the experiment and 0.898 ± 0.26 kg at its end. Egg production did not start during the study period.

A parallel experimental design was applied. Stratified randomization was performed. Group I (*n* = 20) was the negative control group, which included clinically healthy pheasants (without manifestation of IP) fed standard feed (HL-TopMix OOD Company, Kaloyanovo, Sliven, Bulgaria) appropriate for their age. The feed composition and ingredients are resented in Table S1. Group II (*n* = 20) was the experimental group, and this group included pheasants with manifested IP which were fed a diet containing 21 g/kg of the amino acid tryptophan for a period of 30 days. The second experimental group, group III (*n* = 20), consisted of pheasants with manifested IP and received the feed with 10 g/kg of dry extract of milk thistle—silymarin (78% Extr. *Silybum marianum*)—for a period of 30 days. Group IV (*n* = 20) was the positive control group, which contained pheasants with clinical manifestation of IP, fed feed without the tested additives.

The dose of the amino acid L-tryptophan (21 g/kg), used as a feed additive, was selected based on the amounts applied in the experiments with domestic hens exhibiting feather pecking [31,32]. The standardized milk thistle extract, silymarin, was included at 10 g/kg of feed. This dose was selected based on its application in previous studies with birds [33,34].

Behavioral and clinical diagnostics: All 80 pheasants were examined, 20 from each group. To assess and analyze the behavior of the birds, 24 h video surveillance was employed using cameras and a DVR recording device. An ethological study was conducted using ethograms to typify the individual categories of IP: localization and shape. To develop the ethogram protocol, each group of birds was observed for a total of 48 h (using only the light part of the day when the birds were active, that is, six days of eight hours each), with the number of birds performing the corresponding behavior being counted every 15 min in order to determine the entire behavioral complex exhibited by the individuals [35]. The video records were evaluated by two of the researchers and the scores for behavioral changes were determined after discussion between the observers in order to apply uniform criteria when assessing the severity of changes. The data were presented as numerical values for the number of birds performing the studied behavior during the momentary observation. All experimental pheasants were clinically examined and inspected to assess the presence or absence of feather injury (GFP levels), the presence or absence of skin and tissue injury (SFP levels), and the determination of their anatomical localization. The images for the clinical study were captured with a Canon EOS 4000D camera (Canon Bulgaria, Sofia, Bulgaria).

LC-MS/MS determination of serotonin and DA in plasma: One blood sample was taken from 6 male and 6 female birds from each of the four groups of pheasants (*n* = 12). The number of samples was kept to a minimum in order to reduce invasive procedures and stress from the manipulations (Directive 2010/63/EU). The samples were obtained from the superficial wing vein (V. cutanea ulnaris superficialis). They were centrifuged, and the plasma was separated and stored at −80 °C until analysis. The values of the neurotransmitters (serotonin and DA) were determined in plasma using a triple quadrupole mass spectrometer (LC-MS/MS Agilent 6460C, Agilent Technologies, Santa Clara, CA, USA). The extraction and the analytical conditions were set according to the method developed by the authors in [36,37]. The standard curves for serotonin and DA were linear over the following concentration ranges: 1, 10, 25, 50, 100 and 200 ng/mL. Chromatographic separation was performed with a Hypersil GOLD aQ column (2.1 mm inner diameter × 50 mm, Thermo Fisher Scientific, Waltham, MA, USA). The LC-MS/MS apparatus consisted of a 1260 Infinity II quaternary pump and a 1260 Infinity II Vial Sampler and a triple quadrupole mass spectrometer Agilent 6460 with technology Agilent Jet Stream (AJS) (Agilent Technologies, Santa Clara, CA, USA). The mobile phase was 0.1% formic acid in water (A) and 0.1% formic acid in methanol (B) at a flow rate of 0.2 mL/min.

Statistical analysis: Statistical analysis was performed using IBM SPSS Statistics version 26. Group differences in serotonin and dopamine levels were tested with the Kruskal–Wallis H test, followed by Bonferroni-adjusted pairwise comparisons. Differences between sexes were analyzed using the independent samples *t*-test. A significance level of *p* < 0.05 was considered statistically significant.

## 3. Results

### 3.1. Behavioral Diagnostics

The data from the behavioral diagnostics are presented in Table 1. They demonstrate the effect of the addition of TRP and SIL for 30 days on the harmful behavior of pheasants.

The levels of IP in the region of the head, beak and face area of the pheasants (Table 1) showed that the forms of gentle feather pecking (GFP) dominated in the negative control pheasants, while the forms of SFP and tissue pecking were highly expressed in the pheasants of the positive control group. Pecking of the beak and face was most pronounced in the group I pheasants, moderate in the birds from groups II (0.17 ± 0.57) and III (0.08 ± 0.41), and the pheasants of the positive control did not exhibit GFP. Severe pecking in the area of the head was significantly more intense in the pheasants from group IV pheasants compared with the other three groups: the pheasants from group I, which did not exhibit this behavior (*p* < 0.01), the TRP-treated group (*p* < 0.05), and the SIL-treated group (*p* < 0.05).

The levels of IP in the region of the neck of the pheasants are shown in Table 1. Light tissue pecking at the neck was found solely in the pheasants from group I, which was significantly higher in comparison with groups II, III and IV (*p* < 0.01), where the pheasants in these groups did not show this form of GFP. The forms of SFP were not observed in the region of the neck.

The data in the present study, reflecting the IP in the back region of the pheasants (Table 1), showed that the forms of GFP dominated in the negative control pheasants, while the forms of SFP and tissue pecking were strongly expressed in the positive control pheasants in both of the treated groups. Light pecking on the back was found only in the pheasants of group I, it was absent in the other three groups of birds (II, III and IV). SFP in the back region was absent in the pheasants of group I, which was significantly different from the TRP-treated (*p* < 0.001) and SIL-treated (*p* < 0.01) birds and the pheasants with cannibalism in group IV (*p* < 0.001).

IP in the pheasants’ wing region (Table 1) showed that GFP was not detected, while SFP and tissue pecking were highly expressed in the positive control pheasants and in the birds from both of the treated groups. Severe wing pecking was not displayed in the birds of group I, a significantly different finding from the other two groups of pheasants (II and III) treated with supplements (*p* < 0.001) and those of the positive control (group IV, *p* < 0.001).

The data for the IP in the loin area (back) of the pheasants (Table 1) showed that the forms of GFP were observed in the negative control pheasants (group I) and to a minimal extent in the positive control (group IV), but they were absent in both of the treated groups. The forms of SFP and tissue pecking were highly expressed in the positive control pheasants (*p* < 0.001) and the group treated with TRP (*p* < 0.001) or SIL (*p* < 0.05) if compared with the negative control pheasants from group I, in which the absence of these signs was registered. Very intense levels of severe pecking on the loin were exhibited by the pheasants of group IV, compared with those of group I (*p* < 0.001), group II (*p* < 0.05), and group III (*p* < 0.001).

The data from the current study, reflecting the IP in the tail region (Table 1), show that this pecking affects only the tip of the tail feathers. The pheasants from group I (healthy control) did not show tail pecking, but it was strongly expressed in the birds from group IV (*p* < 0.01). In both treated groups II and III, a lower incidence of tail feather pecking was recorded (0.50 ± 1.06) in comparison with group IV (0.75 ± 0.99), although this result was not statistically significant.

The levels of IP in the cloaca region (Table 1) were found in the positive controls only. They were significantly higher in the pheasants from group IV (positive control) compared to the other three groups (*p* < 0.001).

### 3.2. Clinical Examination

**Figure 1:**
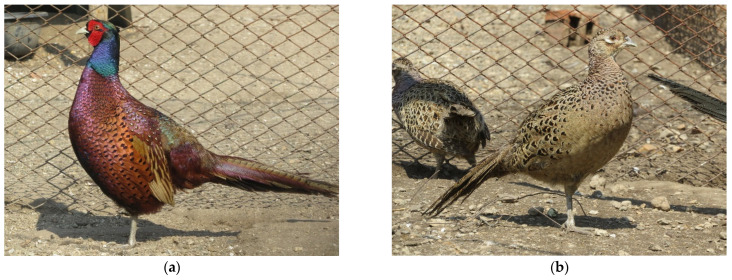
Clinically healthy Black-necked pheasants (*Phasianus colchicus*) without manifestation of cannibalism from group I (**a**) male pheasant; (**b**) female pheasant.

**Figure 2:**
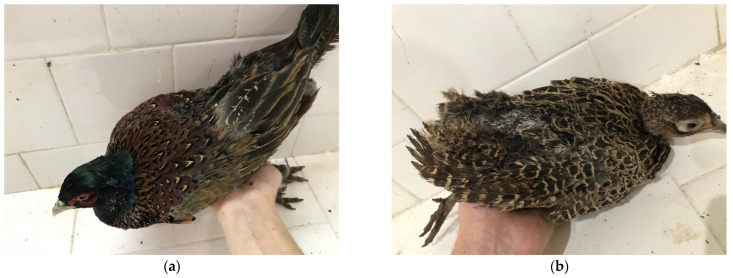
(**a**) Male Black-necked pheasants (*Phasianus colchicus*) with signs of improvement from severe feather pecking and tissue pecking from group II, after treatment with tryptophan; (**b**) female pheasants (*Phasianus colchicus*) with signs of improvement from severe feather pecking and tissue pecking from group II, after treatment with tryptophan.

**Figure 3:**
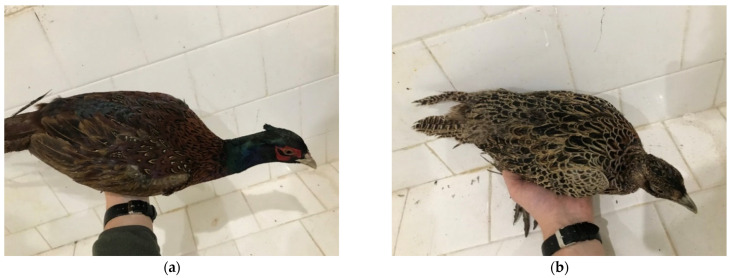
(**a**) Male Black-necked pheasant (*Phasianus colchicus*) with signs of improvement from severe feather pecking and tissue pecking from group III after treatment with silymarin; (**b**) female pheasants (*Phasianus colchicus*) with signs of improvement from severe feather pecking and tissue pecking from group III after treatment with silymarin.

**Figure 4:**
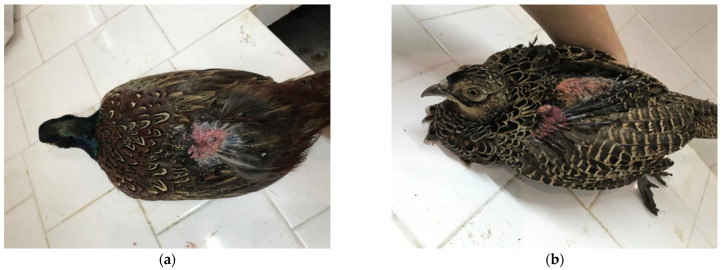
(**a**) Clinical signs of cannibalism in male pheasants from group IV with localization back and tail: severe feather pecking and tissue pecking; (**b**) Clinical signs of cannibalism in female pheasants from group IV with localization back and wings: severe feather pecking and tissue pecking.

**Figure 5:**
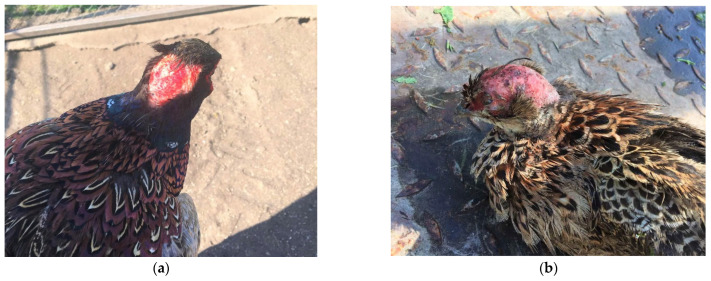
(**a**) Clinical signs of cannibalism in male pheasants from group IV with localization in the head: severe feather pecking and tissue pecking; (**b**) clinical signs of cannibalism in female pheasants from group IV with localization in the head: severe feather pecking and tissue pecking.

**Figure 6:**
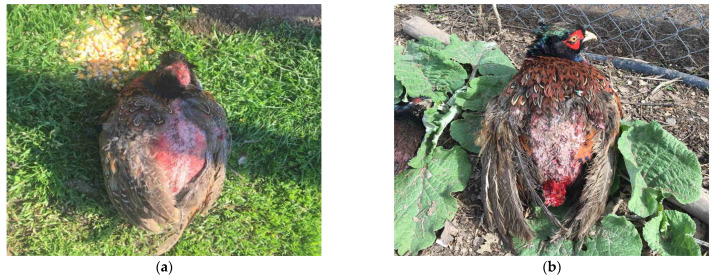
Clinical signs of cannibalism in male pheasants from group IV. (**a**) Severe feather pecking in the region of the head and back, and pecking of the skin and bleeding; (**b**) Severe feather pecking and tissue pecking in the area of the back, rump and tail, accompanied by bleeding and the involvement of deeper tissues.

The data from the clinical investigation are shown in Figure 1, Figure 2, Figure 3, Figure 4, Figure 5 and Figure 6. They demonstrated the effect of TRP and SIL supplementation of the feed for 30 days on the localization and forms of manifestation of cannibalism.

The clinical examination of the pheasants without manifestation of cannibalism from group I (negative control) showed that there were no disorders within the plumage, nor on the surfaces of the face, beak and legs in male (Figure 1a) and female (Figure 1b) birds. The pheasants from group I had a good exterior, their entire plumage cover was preserved, with smooth and shiny feather contours. There were no skin injuries, bare areas of feather loss or breakage and abrasion of the feathers, which was significantly different from the pheasants in the positive control (group IV).

Figure 2 shows the conditions of a male Figure 2a and a female bird Figure 2b from group II, treated with TRP supplementation, after the experiment. A significant improvement in their plumage was observed. The process of feather growth in the affected areas was clearly established, in contrast to the birds in group IV, in which intense and severe pecking was observed. The skin lesions were epithelialized and growth of new feathers was found within the areas of the head, back, rump and wings. The regrowth of the tail feathers was not significant, as their formation and full recovery require a substantially longer period.

A significant improvement in the plumage was observed during the clinical investigation of the pheasants from group III, which were treated with SIL, after the experiment (Figure 3a,b). Full growth and smoothing of the feathers in the affected areas was registered, which differed from the birds in group IV. The skin lesions were regenerated, and new feathers had grown in the affected bare areas of the head, back, rump and wings. The process of tail feather growth had begun, but it is a slow process, as was seen in group II.

The most severe skin and feather lesions were found during the clinical examination in pheasants from the positive control group (group IV). The most frequent localization of skin lesions was found in the areas of the rump (above the tail), back, and wings (Figure 4 in male Figure 4a and female pheasants Figure 4b), which were characterized by the absence of feathers, injured skin and bleeding. Severe injuries were also recorded in the head area (Figure 5a in male and Figure 5b female pheasants) with tissue involvement and the presence of abundant bleeding. SFP was also observed with the formation of bare featherless areas; most often, they were localized around the rump, back (Figure 6a,b), and also on the head (Figure 6a). The bare featherless areas were subjected to tissue pecking by the pheasants and the skin was covered with blood (Figure 6b). If pecking continued, the skin became affected and the injuries extended into deeper tissues and organs, most commonly involving the uropygial gland and the tail (Figure 6b). Often, several forms (simultaneous FP or tissue pecking) were observed in the same individuals and in several different anatomical areas (Figure 4, Figure 5 and Figure 6).

### 3.3. Neurobiological Studies

The levels of the neurotransmitters serotonin and DA in plasma are shown in Table 2, and the individual concentrations are shown in Tables S2 and S3.

The Kruskal–Wallis test showed a significant effect of group membership on serotonin levels (H = 36.723, df = 3, *p* < 0.001). Post hoc pairwise comparisons using Bonferroni correction indicated that group I had significantly higher serotonin levels than group IV (*adj. p* = 0.000) and group III (*adj. p* = 0.000). Group II (TRP treated) also differed significantly from the birds with IP from group IV (*adj. p* = 0.001) and group III (SIL treated, *adj. p* = 0.028). No significant difference was observed between the birds in group I (negative control) and group II (*adj. p* = 0.653), nor between those in group III and group IV (*adj. p* = 1.000).

Similarly, the dopamine levels differed significantly across the groups (H = 17.175, df = 3, *p* = 0.001). Bonferroni-adjusted pairwise comparisons revealed a significant difference between group I and group IV (*adj. p* = 0.001), as well as between group III and group IV (*adj. p* = 0.010). All other comparisons were not statistically significant. For example, the difference between group I and group II (*adj. p* = 0.692) and between group II and group III (*adj. p* = 1.000) did not reach significance.

## 4. Discussion

Gentle pecking of the head area—the face and beak—was observed in healthy pheasants, in contrast to those manifesting cannibalism. Many authors believe that this type of pecking is a cognitive form of social behavior, most often observed at a young age [6,38,39], which causes no damage to either the birds’ plumage or their skin [40,41,42].

A severe form of pecking in the head area id strongly expressed in pheasants displaying cannibalism, unlike that observed in healthy birds. It is important to discriminate the aggressive behavior from IP. Birds who perform aggressive pecking aims to establish a hierarchy in the flock [11,43], while IP is a non-aggressive manifestation, and rather is a type of deviant behavior [44,45,46]. The application of SIL and TRP reduced SFP and tissue pecking in the head area in pheasants with cannibalism. In healthy pheasants, GFP was observed in the neck area, while diseased pheasants did not demonstrate this FP type.

The forms of GFP in the back region predominated in healthy pheasants, while the SFP and tissue pecking forms were strongly expressed in pheasants manifesting behavioral disorders. Gentle pecking on the back was observed only in clinically healthy pheasants, without severe pecking episodes. The opposite was observed in pheasants with cannibalism, which exhibited very high levels of severe pecking with the absence of gentle pecking on the back. Again, this can be attributed to the fact that the GFP, under certain conditions, could progress into SFP and tissue pecking [14,42,47]. Our data are consistent with the enhanced SFP manifestations reported in laying hens [5,16,38]. After dietary treatment with TRP and SIL, the pheasants demonstrated a slight decrease in SFP frequency in this area.

In pheasants, only the severe form of wing pecking was detected. The extent of SFP and tissue pecking in the wing area was high in birds with behavioral disorders and was not manifested in healthy pheasants. Previous studies have reported severe injuries in the wing area of hens as a result of cannibalism [10,15,48]. Although the difference was not statistically significant, treatment with TRP and SIL was associated with a slight decrease in IP in the wing area, which was likely due to the substantially greater difficulty of controlling major and more severe pecking types compared with GFP. This is explained by the fact that under certain conditions, GFP can progress into a severe form, as reported by a number of researchers of hens [14,39,47].

The GFP in the area of the rump/tail base predominated in healthy pheasants, while the SFP and tissue pecking forms were highly expressed in birds with cannibalism. Gentle rump pecking was demonstrated by clinically healthy pheasants, while severe pecking in this area was not observed. The opposite was found in pheasants with cannibal behavior, which showed very high levels of severe pecking, while the gentle rump area pecking was only minimal. Similarly to the abovementioned data, it is possible that the gentle form (specific to healthy birds) has progressed into severe pecking in birds with cannibalism due to factors and errors related to game birds’ rearing. In poultry, GFP episodes in young chicks have evolved into SFP in mature hens [49,50,51]; similar findings were reported in a study between male and female domestic turkeys [11]. Pheasants with SFP in the rump area exhibited reduced IP type behavior after treatment with SIL, indicating the powerful effect of milk thistle extract on the behavior of game birds. The amino acid TRP also significantly reduced SFP levels in the sacral area of pheasants due to the action of TRP as a precursor in the production of serotonin and DA in birds [52,53].

A specific type of tail feather pecking was found in the pheasants. This is explained by the fact that pheasants are a species with very long tail feathers, meaning that these feathers could easily become the target of pecking. Tail feather pecking has also been observed in chickens [10,54,55]. Clinically healthy pheasants did not demonstrate tail pecking, while the tail feathers of birds with cannibal behavior were severely broken or completely missing. The evaluation of the tail pecking level is more specific because birds mainly peck the feather tips, e.g., break the feather [12,41,56], while they could peck the feathers at the tail base only when the tail grows, resulting in completely absent feathers. After the supplementation of TRP and SIL to pheasants, the tail pecking levels decreased but this result was statistically significant only between group IV and group I. In pheasants with lacking tails, the tail feathers had started to regrow after the supplementation period.

A separate, specific form of cannibalism in pheasants was the cloacal pecking described in hens [11,38,57]. Cloacal pecking was observed only in pheasants with cannibalism. This type of cannibalism also appeared in birds with good plumage status [41,46] and was more often described at the beginning of the egg-laying period in hens [15,41]. After the administration of TRP and SIL supplements, the pheasants did not demonstrate cloacal pecking. In addition to the beneficial effect of the dietary supplements, this may also be due to the fact that unlike other types of severe pecking, cloacal pecking was not as intensively manifested and thus was easier to correct.

Numerous studies have affirmed that the neurobiological control of cannibalism in birds is related to changes in serotonin and DA levels [53,58]. Aromatic amino acids (TRP, TYR and PHE) are precursors for the synthesis of serotonin and DA. The neurobiological mechanisms that govern IP behavior in birds are associated with changes in serotonin- and dopamine-mediated transmission of nerve impulses in the brain and the hypothalamic–pituitary–adrenal axis [10,52]. It has been established that central serotonin and DA synthesis was influenced by the presence of their AAAs precursors in the blood plasma, which cross the blood–brain barrier and increase their levels [9,12,59]. Serotonin activity plays a key role in the physiological characteristics of IP [54,60]. The distribution of serotonin fibers in the avian brain controls the influence of serotonin on fear and social behavior and thus on IP manifestation [61,62]. In the present study, blood samples were not collected from all pheasants involved in the ethological and clinical studies because of the implementation of ethical standards of work with these wild bird species, limiting unnecessary invasive procedures and stress, according to Directive 2010/63/EU. However, on the basis of a smaller number of samples, the data from the blood tests were sufficient to identify the main trends and confirm the observed behavioral differences between the control and tested birds. In pheasants with pronounced manifestations of cannibalism, serotonin levels were significantly lower than those in clinically healthy game birds of the same species. In support of this, a number of authors have described lower blood serotonin in hens with FP in relation to those without feather pecking [62,63]. After the dietary supplementation of high concentrations of TRP (21 g/kg) to pheasants exhibiting IP, a marked increase in serotonin levels was found. Similarly, feeding higher doses of TRP (from 2.6 to 22.6 g/kg) to growing bantam chickens resulted in the suppression of SFP in adulthood [32]. A diet with a very high TRP content (21 g/kg) in contrast to the standard one (1.6 g/kg), led to a reduced level of GFP in chickens [64]. A similar finding regarding reduced FP behavior has been shared by other scientists after the application of higher dietary levels of TRP [31,60]. The application of SIL was not able to significantly change blood serotonin levels vs. control groups of pheasants with cannibalism.

Dopamine transmission of nerve impulses in the brain also plays a key role in the neurophysiological features of IP [31,32]. Dopamine improves the ability to cope with fear and stress, resulting in reduced levels of IP and cannibalism [41,65]. Similar data were reported in terms of DA levels. For example, pheasants with pronounced IP had lower DA levels than clinically healthy birds of the same species. In published studies, direct DA regulation of FP and cannibalism has been shown in hens [31,52,54]. Furthermore, it has been demonstrated that higher DA levels can improve the stress-coping ability of birds, resulting in reduced FP levels [41,65]. Many researchers have reported lower plasma DA levels in chickens with cannibalism compared with healthy ones [52,60,61]. Increased plasma DA levels were found after the administration of high doses of TRP (21 g/kg) to pheasants exhibiting cannibalism in support of the effect of this AAA, a precursor for the synthesis of monoamine neurotransmitters serotonin and DA, responsible for the regulation of IP in hens [9,53,59]. The restoration of DA under the influence of silymarin treatment can be attributed to its antioxidant and neuroprotective effects [22,23,24,25,26].

## 5. Conclusions

The data obtained from the present study allowed us to conclude that in clinically healthy game birds, the gentle FP type predominated as a motivation for social and cognitive behavior, without serious injury to the plumage. Severe forms of FP and tissue pecking were observed in pheasants with clinical manifestations of cannibalism, they seriously damaged the plumage and tissues of other birds. As a result of stress factors, improper diet, and being raised in captivity, the gentle FP type specific to healthy game birds evolved into a severe form in individuals with cannibalism. The administration of tryptophan and silymarin led to a significant reduction in IP levels in the studied game birds. For the first time, the exact localization of the gentle and severe IP types has been established in Black-necked pheasants. In healthy pheasants, low intensity GFP, localized in the head area (face and beak), neck, back, and rump was observed. In pheasants with obvious cannibalism, SFP and high-intensity tissue pecking were observed in the following anatomical areas: head, back, wings, and rump, along with the specific forms: pecking of the tail tips and cloacal cannibalism. Despite these findings, further studies are required to evaluate the long term effects of treatment with tryptophan and silymarin on severe forms of FP and tissue pecking.

The blood levels of monoamine neurotransmitters (serotonin and DA) in pheasants with cannibalism were significantly lower than those in clinically healthy birds within the same species. The addition of tryptophan to the diet of birds manifesting cannibalism caused a substantial increase in plasma serotonin and a tendency towards a moderate increase in their DA concentrations. One of the limitations of this study is related to the number of samples used for the dopamine and serotonin evaluation. Investigating the levels of these neurotransmitters in a larger population will shed more light on the kinetics of both compounds in pheasants with behavioral disorders.

## Figures and Tables

**Table 1:** Ethogram reflecting injurious pecking in Black-necked pheasants (*Phasianus colchicus*) (*n* = 20) in the negative control (group I), those fed feed containing 21 g/kg of tryptophan (TRP, group II), 10 g/kg of silymarin (SIL, group III), and the positive control (group IV).

Anatomical Area	I Negative Control	II Treated with TRP	III Treated with SIL	IV Positive Control
Forms of gentle feather pecking (GFP) (number of birds performing the behavior)				
Face and beak	0.33 ± 0.96	0.17 ± 0.57	0.08 ± 0.41	0.0 ± 0.0
Neck	0.50 ± 1.22	0.0 ± 0.0 ^1b^	0.0 ± 0.0 ^1b^	0.0 ± 0.0 ^1b^
Back	0.25 ± 0.90	0.0 ± 0.0	0.0 ± 0.0	0.0 ± 0.0
Wings	behavior was not performed			
Rump	0.58 ± 1.61	0.0 ± 0.0	0.0 ± 0.0	0.25 ± 1.23
Forms of severe feather pecking (SFP) and tissue pecking (cannibalism) (number of birds performing the behavior)				
Head	0.0 ± 0.0	0.25 ± 0.68	0.25 ± 0.90	0.58 ± 0.93 ^1b, 2a, 3a^
Neck	behavior was not performed			
Back	0.0 ± 0.0	1.75 ± 1.80 ^1c^	1.50 ± 1.98 ^1b^	1.92 ± 2.00 ^1c^
Wings	0.0 ± 0.0	1.42 ± 1.72 ^1c^	1.29 ± 1.27 ^1c^	1.67 ± 1.93 ^1c^
Rump	0.0 ± 0.0	2.42 ± 2.34 ^1c^	1.42 ± 1.82 ^1a^	4.04 ± 2.37 ^1c, 2a, 3c^
Tail	0.0 ± 0.0	0.50 ± 1.06	0.50 ± 1.06	0.75 ± 0.99 ^1b^
Cloaca	0.0 ± 0.0	0.0 ± 0.0	0.0 ± 0.0	0.50 ± 1.06 ^1c, 2c, 3c^

^a^—*p* < 0.05; ^b^—*p* < 0.01; ^c^—*p* < 0.001: ^1^—compared with group I; ^2^—compared with group II; ^3^—compared to group III.

**Table 2:** The data for serotonin and dopamine levels in Black-necked pheasants (*Phasianus colchicus*) (*n* = 12) in the negative healthy control (group I), those fed feed containing 21 g/kg of TRP (group II) or 10 g/kg of SIL (group III), and the positive control (group IV).

Group	Serotonin (ng/mL)	Dopamine (DA, ng/mL)
I, negative control	237.77 ± 17.01 ^a^	0.67 ± 0.23 ^a^
II, treated with TRP	155.48 ± 14.45 ^a^	0.56 ± 0.32 ^a, b^
III, treated with SIL	80.15 ± 06.96 ^b^	0.62 ± 0.25 ^a^
IV, positive control	60.51 ± 08.02 ^b^	0.33 ± 0.10 ^b^
*p*-value	<0.001	<0.001

Different letters indicate statistically significant differences between groups in terms of serotonin or dopamine concentrations at *p* < 0.05.

## Data Availability

Data are contained within the article or Supplementary Materials.

## Supplementary Materials

The following supporting information can be downloaded at: https://www.mdpi.com/article/10.3390/ani15243561/s1, The feed fed to pheasants is shown in Table S1: Complete basal diet fed to Black-necked pheasants (*Phasianus colchicus*). Primary data used in Section 3.3. Neurobiological studies are provided, these are the blood samples obtained from 12 birds from each group (total 48 pheasants), used for analysis of plasma levels of neurotransmitters (serotonin and DA). Table S2: Primary data on levels of Serotonin (ng/mL) in Black-necked pheasants (*Phasianus colchicus*) (*n* = 12). Table S3: Primary data on levels of Dopamine (DA, ng/mL) in Black-necked pheasants (*Phasianus colchicus*) (*n* = 12).

## Abbreviations

The following abbreviations are used in this manuscript:

EU	European Union
IP	Injurious pecking
PF	Feather pecking
AAAs	Aromatic amino acids
TRP	Tryptophan
PHE	Phenylalanine
TYR	Tyrosine
SFP	Severe feather pecking
SIL	Silymarin
5-HT	5-hydroxytryptamine
DA	Dopamine
LC/MS	Liquid chromatography-mass spectrometry
DVR	Digital video recorder
AJS	Agilent jet Stream
GFP	Gentle feather pecking
adj. *p*	Adjusted *p* value.
