# Supplementary material for: Ethological, Clinical, and Neurobiological Studies on Cannibalism in Black-Necked Pheasants (Phasianus colchicus) and Correction of Behavioral Disorders by Applying Nutritional Supplements
Source: Animals (Basel). 2025 Dec 11;15(24):3561. doi: 10.3390/ani15243561 (PMC12729755; doi:10.3390/ani15243561)
Supplement: Supplementary file 1 [file animals-15-03561-s001.zip › animals-4022013-supplementary.pdf]

Table S1. Complete basal diet fed to Black-necked pheasants (Phasianus colchicus)

**Feed ingredients**	Basal diet for **Group I** and **Group II**	Tryptophan supplemented **group II**	Silymarin supplemented **group III**
**Composition**			
Corn, Sunflower meal, Wheat, Calcium carbonate, Corn gluten meal, Soybean meal (GM soybean for EU), Dried corn distillers' mash, Sugar beet molasses, Crude sunflower oil, Monocalcium phosphate, L – lysine, Sodium chloride, Premix, Methionine hydroxy analogue, Toxin binder, Betaine, Antioxidant, Enzyme complex - not used			
**Analytical composition**			
Moisture	10.0%	10.0%	10.0%
Crude protein	16.5%	16.5%	16.5%
Crude fiber	5.4%	5.4%	5.4%
Crude fat	3.6%	3.6%	3.6%
Crude ash	13.2%	13.2%	13.2%
Calcium	3.99%	3.99%	3.99%
Phosphorus	0.66%	0.66%	0.66%
Sodium	0.13%	0.13%	0.13%
Lysine	0.82%	0.82%	0.82%
Methionine	0.39%	0.39%	0.39%
**Nutritional supplements**			
Vitamin A	10 000 IU/kg	10 000 IU/kg	10 000 IU/kg
Vitamin D3	3200.0 IU/kg	3200.0 IU/kg	3200.0 IU/kg
Vitamin E	15 mg/kg	15 mg/kg	15 mg/kg
Betaine (anhydrous betaine)	112 mg/kg	112 mg/kg	112 mg/kg
Iron (ferrous sulfate monohydrate)	131.5 mg/kg	131.5 mg/kg	131.5 mg/kg
Manganese (manganous oxide)	104.2 mg/kg	104.2 mg/kg	104.2 mg/kg
Zinc (zinc oxide)	69.8 mg/kg	69.8 mg/kg	69.8 mg/kg
Copper (copper (II) sulfate pentahydrate)	17.4 mg/kg	17.4 mg/kg	17.4 mg/kg
Iodine (calcium iodide)	0.95 mg/kg;	0.95 mg/kg;	0.95 mg/kg;
Selenium (sodium selenite)	0.35 mg/kg	0.35 mg/kg	0.35 mg/kg
Tryptophan	0.4%	**21 g/kg**	0.4%
Lysine	0.29%	0.29%	0.29%
Methionine	0.09%	0.09%	0.09%
Bentonite	432.500 mg/kg	432.500 mg/kg	432.500 mg/kg
BHT (E321)	4.40 mg/kg	4.40 mg/kg	4.40 mg/kg
Propyl gallate (E310)	2.20mg/kg	2.20mg/kg	2.20mg/kg
6 Phytase	500 units/kg	500 units/kg	500 units/kg
Endo-1.4-beta-xylanase	1650.0IU/kg	1650.0IU/kg	1650.0IU/kg
**Additional supplements**			
Silymarin	-	-	**10 g/kg**

Table S2. Primary data on levels of Serotonin (ng/ml) in Black-necked pheasants (*Phasianus colchicus*) (n=12).

Negative control	Tryptophan supplemented	Silymarin supplemented	Positive control
263.17	145.52	91.10	46.05
107.61	157.14	47.50	81.39
329.53	165.82	68.96	94.73
294.74	147.32	57.26	58.06
213.68	105.20	61.22	17.23
209.35	125.13	87.74	43.49
267.32	107.41	107.64	12.33
189.07	291.07	126.76	76.02
259.47	155.67	104.89	71.78
199.76	110.77	57.15	46.99
286.25	167.75	79.71	86.95
233.27	186.95	71.89	91.06

Table S3. Primary data on levels of Dopamine (DA, ng/ml) in Black-necked pheasants (*Phasianus colchicus*) (n=12).

Negative control	Tryptophan supplemented	Silymarin supplemented	Positive control
0.49	0.44	0.34	0.23
0.67	0.29	0.70	0.27
0.59	0.54	0.24	0.27
1.15	0.38	0.66	0.33
0.48	0.43	0.85	0.53
0.53	0.45	0.37	0.22
0.56	1.280	0.52	0.43
1.05	0.611	0.74	0.25
0.60	1.175	0.42	0.42
0.53	0.499	0.61	0.39
0.89	0.386	0.87	0.25
0.46	0.270	1.09	0.41
